# Relationship between matrix metalloproteinases and the occurrence and development of ovarian cancer

**DOI:** 10.1590/1414-431X20176104

**Published:** 2017-05-18

**Authors:** Y. Zhang, Q. Chen

**Affiliations:** Department of Obstetrics and Gynecology, the Second Affiliated Hospital of Nanchang University, Nanchang, China

**Keywords:** Ovarian cancer, Matrix metalloproteinases (MMPs), Tissue inhibitor of metalloproteinases (TIMPs), Invasion, Metastasis

## Abstract

Ovarian cancer is one of the most malignant genital cancers, with a high mortality rate. Many researchers have suggested that matrix metalloproteinases (MMPs) have remarkably high expression in ovarian cancer tissues. MMPs are considered to be related to the occurrence, development, invasion and metastasis of ovarian cancer. Moreover, some studies have discovered that the unbalance between MMPs and tissue inhibitor of metalloproteinases (TIMPs) are associated with the malignant phenotype of tumors. This review summarizes the latest research progress of MMPs in ovarian cancer. The investigation of MMP mechanism in ovarian cancer will facilitate the development of effective anti-tumor drugs, and thereby improve the survival rate of patients with ovarian cancer.

## Introduction

Ovarian cancer is the third most common malignancy of female genitalia, and its etiology is still uncertain. The incipient symptoms of ovarian cancer are not obvious, and therefore patients are often in the middle-late stages of the disease when diagnosed. Moreover, it is difficult to thoroughly remove the tumor mass by surgery. Thus, ovarian cancer is refractory and difficult to treat, especially in patients with early metastasis. Ovarian cancer is the deadliest gynecologic tumor and seriously threatens women’s health and lives. Therefore, in order to timely and effectively diagnose and treat ovarian cancer, as well as to improve the prognosis and survival rate of patients, it is essential to identify ovarian cancer-specific tumor markers with high sensitivity. Herein, the latest research progress of matrix metalloproteinases (MMPs) in ovarian cancer is reviewed.

## MMPs

**Figure 1.**
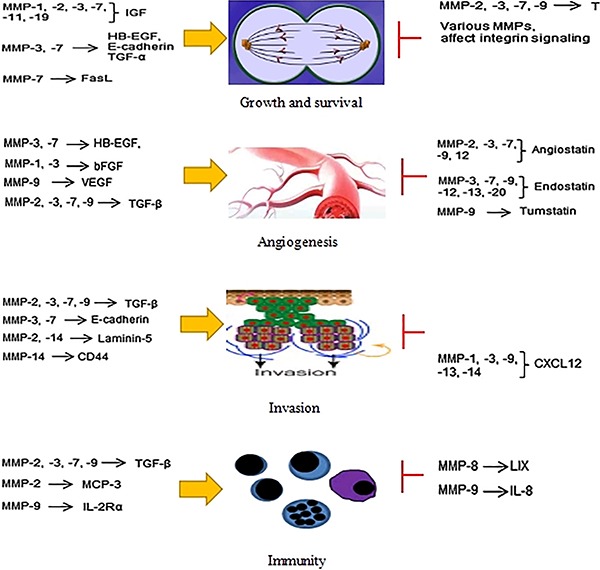
Important effects of matrix metalloproteinases (MMPs) in cancer progression. MMPs play important roles on central aspects of tumor progression, such as tumor growth and survival, angiogenesis, invasion, and modulation of the immune response.

Numerous studies have shown that the occurrence, development, invasion and metastasis of ovarian cancer are closely associated with MMPs. MMPs are a large family of calcium-dependent zinc-containing endopeptidases, which are typically composed of five structural zones: 1) pro-peptide, which maintains the latency of the proenzymes; 2) catalytic domain, including zinc-ion binding site; 3) hydrophobic signal peptide; 4) hemopexin-like C-terminal domain, which is associated with the substrate specificity; 5) hinge region, containing prolines. Collectively, MMPs are capable of degrading a variety of component proteins in extracellular matrix (ECM), such as collagen, elastin, gelatins, and casein (1). ECM, consisting of basement membrane and stroma cells, is an important regulatory barrier for tumor metastasis. MMPs degrading actions in ECM lead to changes in its structure and the expression of its cellular surface receptors, which prompts the occurrence, development, invasion and metastasis of malignant tumors. To date, the MMP family encompasses 25 related members. Based on the differences in their structure organization and substrate specificity, MMPs can been divided into 6 groups (2): 1) collagenases (MMP-1, 8, 13, and 18), of which the main function is degrading collagen types I, II and III; 2) gelatinases (MMP-2 and MMP-9), mainly acting on denaturing and cleaving type IV collagen and gelatin; 3) stromelysins (MMP-3, 7, 10, 11, 26 and 27), displaying hydrolysis ability of a broad range of ECM proteins, such as collagen types III, IV, V, elastin, proteoglycans and glycoprotein; 4) elastases (MMP-12); 5) membrane-type specific MMPs (MMP-14, 15, 16, 17, 24, 25) with a furin cleavage site in the pro-peptide region that are mainly related to the activation of MMP-2; and 6) other MMPs (MMP-19, 20, 21, 22, 23, and 28). Studies have reported that MMPs not only play an important role in embryonic development, wound healing and organization remodeling, but also regulate the occurrence and development of malignant tumors by participating in cellular processes of proliferation, apoptosis and angiogenesis (Figure 1). Bourboulia and Stetler-Stevenson (3) reported that MMPs lead to the invasion and metastasis of tumor cells via migration through stroma.

## Relationship of MMPs with ovarian cancer

MMP-1, also known as interstitial collagenase, is the most commonly expressed enzyme among the members of MMPs family. It is widely spread in mesenchymal, endothelial and epithelial cells. The gene encoding MMP-1 is localized in chromosome 11q22-q23. Overexpression of MMP-1 has been implicated in various malignant tumors and found to be closely linked to early metastasis and poor prognosis, especially lymph node cancer. The association of MMP-1 with the migration and invasion of tumor cells involves the G protein-coupled receptor, protease-activated receptor-1 (PAR1). Agarwal et al. (4) have demonstrated that MMP-1 activates PAR1, which is an important signal transducer in angiogenesis and metastasis in peritoneal mouse model of ovarian cancer. The activation of MMP1-PAR1 induces the secretion of angiogenic factors of interleukin 8 (IL-8) and growth-regulated oncogene-alpha from ovarian carcinoma cells, which act on endothelial CXCR1/2 receptors in a paracrine manner, leading to endothelial cell proliferation, tube formation and migration. Therefore, MMP1-PAR1-CXCR1/2 paracrine pathways have been suggested as new targets for ovarian cancer therapy. Wang et al. (5) have demonstrated that the increased expression level of MMP-1 is closely correlated with the increased invasion of epithelial ovarian cancer (EOC) cells, which is mediated by PAR1. Moreover, both MMP1 and PAR1 are initially induced by lysophospholipid acid (LPA) in the process of invasion and metastasis in EOC. It has been suggested that MMP1-PAR1 axis might be a target for inhibition of invasion and metastasis in ovarian cancer. MMP-1 expression in ovarian cancer cell lines also requires other regulators, such as mixed lineage kinase 3 (MLK3). Zhan et al. (6) reported that MLK3 is a mitogen-activated protein kinase kinase kinase (MAP3K) that activates MAPK signaling pathways and regulates cellular responses such as proliferation, migration and apoptosis. High levels of total and phospho-MLK3 in ovarian cancer cell lines have been reported. Additionally, MLK3 is also required for the invasion in ovarian cancer cell lines SKOV3 and HEY1B.

The relative molecular mass of MMP-2 is 72 kDa. *MMP-2* gene is mapped in the 16q21 chromosome region, and is composed of 13 exons and 12 introns. MMP-2 mainly degrades type IV collagen, which is the primary component of the ECM and basement membrane. Since the invasion and metastasis of tumors largely depend on the degradation of ECM, expression of MMP-2 plays a positive role in the progression of ovarian cancer. Overexpression of MMP-2 in peritoneal implants of ovarian cancer cells is related to a significant risk of death (7). The expression of MMP-2 in cystic fluid of ovarian serous cancer and mucous cancer is significantly higher compared with benign and borderline ovarian tumor (8). Consistently, Huang and Sui (9) showed that the positive rate of MMP-2, vascular endothelial growth factor C (VEGF-C) and E-cadherin in ovarian cancer is higher than that in benign and borderline ovarian tumors. MMP-2 level is positively correlated with clinical stage and metastasis of ovarian cancer, but not with pathologic grading and age in ovarian cancer patients (10). Tumor-derived MMP-2 expression predicts a lower overall survival rate and therefore might be an independent prognostic factor for ovarian cancer (11). Several studies have attempted to explore the role of MMP-2 as a therapeutic target for ovarian cancer. For instance, Wang et al. (12) showed that epigallocatechin-3-gallate inhibits the proliferation and migration of ovarian cancer cells by down-regulation of MMP-2 expression. Estradiol-induced migration and invasion in SKOV3 cells is mediated by the regulation of MMP-2 expression (13). Catalpol suppressed proliferation and accelerated apoptosis of OVCAR-3 ovarian cancer cells via promoting microRNA-200 expression and reducing MMP-2 signaling (14). Zoledronic acid exerts robust inhibitory activity on cell invasion of HeyA8-MDR and OVCAR-5 ovarian cancer cells through decreasing the intracellular level of MMP-2 (15).

Known as matrilysin-1, MMP-7 is the smallest member in the MMP family. The encoding gene is localized on chromosome 11q21-q22. MMP-7 is composed of a pro-peptide and a catalytic domain, and has hydrolase activity towards a wide range of ECM proteins, such as elastin, fibronectin, proteoglycan, collagen, laminin and casein (16). Abnormal MMP-7 overexpression is detected in cancer cells, and plays a crucial role in both early and later stages of tumors (17). Wang et al. (18) have shown that overexpression of MMP-7 in EOC is largely stimulated by VEGF and IL-8. Moreover, MMP-7 promotes the invasion and metastasis of ovarian cancer cells by activating gelatin enzyme. MMP-7 also promotes the invasion and metastasis of ovarian cancer through mesothelin (MSLN)-activated MAPK/ERK and JNK pathways (19). Therefore, blocking the MSLN-related pathway might be a potential therapeutic measure for the inhibition of ovarian cancer progression. Yoshioka et al. (20) reported that WNT7A is absent in normal ovary, and in borderline and benign ovarian tumors, but overexpressed in the epithelium of ovarian serous carcinomas. While overexpression of WNT7A stimulates MMP-7 promoter, mutation of TCF binding site in MMP7 promoter indicates that activation of MMP-7 promoter by WNT7A was mediated by β-catenin/TCF signaling. Zhao et al. (21) have shown that triptolide inhibits the migration and invasion of ovarian cancer cells through suppressing the expression of MMP-7 *in vitro*, and thus concluded that triptolide can be used as a fine candidate for treatment of ovarian cancer. Increased metabolites of 5-lipoxygenase from hypoxic ovarian cancer cells can facilitate the migration and invasion of macrophages, which is achieved via up-regulation of MMP-7 expression through p38 pathway (22).

MMP-9 belongs to the type IV gelatin enzyme, with relative molecular mass of 92 kDa. MMP-9 is capable of degrading type IV collagen of the basement membrane, which plays an essential role in the invasion and metastasis of malignant tumors. Wang et al. (23) demonstrated that platelet-derived growth factor-D promotes the invasion and metastasis of ovarian cancer through up-regulating the expression of MMP-9. Hu et al. (24) have found that MMP-9 level in ovarian cancer is higher compared with normal ovarian tissues and benign ovarian tumors. Furthermore, the postoperative levels of MMP-9 in ovarian cancer are significantly reduced compared with preoperative levels. Consequently, MMP-9 has been suggested as a potential serum marker for ovarian cancer diagnosis, and high serum MMP-9 level might be a predictor for refractory tumors. Further, Hu et al. (24) have shown that MMP-9 siRNA reduces the invasion and adhesion ability of ovarian cancer HO8910PM cells. MMP-9 expression is positively correlated with differentiation degree, FIGO staging and lymph node metastasis phase in ovarian cancer, but not with tissue types, suggesting that increased MMP-9 expression is associated with poor prognosis of ovarian cancer (25). MMP-9 has been suggested as a potential therapeutic target for ovarian cancer therapy. It has been shown that filamin B can suppress the growth and metastasis of human ovarian cancer by down-regulating the activity of MMP-9 and secretion of VEGF-A (26). More recently, therapies targeting one or more MMPs have also been proposed. Wang et al. (27) reported that Fe-MIL-101 suppresses the proliferation of human SKOV3 ovarian cancer cells via downregulation the expression of MMP-2 and -9. Bisdemethoxycurcumin can also inhibit ovarian cancer cells via reducing oxidative stress-mediated expression of MMP-2 and -9 (28).

Evidence has been reported supporting the strong association between other members of the MMPs family and the occurrence, development, and metastasis of ovarian cancer (29). Qiu et al. (13) found that the regulation of MMP-3 and MMP-2 expression is involved in the estradiol-induced migration and invasion of SKOV3 cells. Phorbol-12-myristate 13-acetate, an activator of the PKC pathway, may increase the expression level of MMP-7 and MMP-10 in SKOV3 cells (30). MMP-8 can promote the invasion and metastasis of ovarian cancer cell via up-regulation of IL-1β (31). It has been suggested that MMP-12 82 A/G polymorphism may increase the susceptibility of ovarian cancer despite not being significantly associated with overall cancer risk (32,33). ETS-1 protein can promote MMP-13 expression in SKOV3 cells by regulation of VEGF (34). MMP-14, also known as membrane-type 1 MMP (MT1-MMP), plays an important role in the invasion and metastasis of a variety of cancers by activation of pro-MMP-2 and ECM degradation (35). Higher expression of MMP-14 is associated with lower progression and better prognosis of ovarian carcinoma (36). Moreover, patients with double expression of MMP-14 and CD44 have a poor prognosis (37). Kaimal et al. (38) have shown that a monoclonal antibody, which selectively blocks MMP-14, can restrain the growth, invasion and angiogenesis of ovarian cancer. Human leukocyte antigen G-G is associated with the invasion and metastasis in tumor through mediating MMP-15 expression in ovarian cancer cells (39). The roles of MMPs in ovarian cancer published since 2010 are summarized in Table 1.

**Table 1.** An overview of studies on the roles of matrix metalloproteinases (MMPs) in ovarian cancer published since 2010.

Author, year	MMP molecule(s)	Role in ovarian cancer
Pei et al., 2016 (28)	MMP-2 and MMP-9	Bisdemethoxycurcumin inhibits ovarian cancer cells via reducing oxidative stress-mediated expression of MMP-2 and -9.
Wang et al., 2016 (27)	MMP-2 and MMP-9	Fe-MIL-101 suppresses the proliferation of human SKOV3 ovarian cancer cells via downregulation the expression of MMP-2 and -9.
Vos et al., 2016 (37)	MMP-14	Patients with double expression of MMP-14 and CD44 have a poor prognosis.
Fu et al., 2015 (11)	MMP-2	Tumor-derived MMP-2 expression predicts a lower overall survival rate and could be an independent prognostic factor in patients with ovarian cancer.
Gonzalez-Villasana et al., 2015 (15)	MMP-2	Zoledronic acid exerts robust inhibitory activity on cell invasion of HeyA8-MDR and OVCAR-5 ovarian cancer cells through decreasing the intracellular level of MMP-2.
Liu etal., 2015 (33)	MMP-12	MMP-12 82 A/G polymorphism increases the susceptibility to ovarian cancer despite not being significantly associated with overall cancer risk.
Chen et al., 2015 (32)	MMP-12	MMP-12 82 A/G polymorphism is a genetic risk factor for epithelial ovarian carcinoma.
Gao et al., 2014 (14)	MMP-2	Catalpol suppresses proliferation and accelerated apoptosis of OVCAR-3 ovarian cancer cells via promoting microRNA-200 expression and reducing MMP-2 signaling.
Bandaru et al., 2014 (26)	MMP-9	Filamin B (FLNB) suppresses the growth and metastasis of human ovarian cancer by down-regulating the activity of MMP-9 and secretion of vascular endothelial growth factor-A (VEGF-A).
Trudel et al., 2014 (36)	MMP-14	Higher expression of MMP-14 is associated with lower progression and better prognosis of ovarian carcinoma.
Wang et al., 2013 (10)	MMP-2	MMP-2 plays a positive role in the invasion and metastasis of ovarian cancer.
Al-Alem et al., 2013 (30)	MMP-7 and MMP-10	Targeted inhibition of MMP-7 and MMP-10 may provide potential ovarian cancer therapeutic strategy.
Li et al., 2013 (25)	MMP-9	Increased expression of MMP-9 is associated with poor prognosis in ovarian cancer.
Kaimal et al., 2013 (38)	MMP-14	MMP-14-dependent invasion and metastasis is effectively inhibited by intraperitoneal administration of monoclonal MMP-14 antibody.
Zhao et al., 2012 (21)	MMP-7	Triptolide inhibits the migration and invasion of ovarian cancer cells by suppression of MMP-7.
Chang et al., 2012 (19)	MMP-7	Mesothelin enhances ovarian cancer invasion by MMP-7 expression through the MAPK (mitogen-activated protein kinase)/ERK (extracellular-signal-regulated kinase) and JNK (c-Jun N-terminal kinase) signal transduction pathways.
Ghosh et al., 2012 (34)	MMP-9 and MMP-13	Activation of PI3K/AKT (phosphatidylinositol-3-kinase) and p38 MAPK by VEGF activates MMP-9 and MMP-13, leading to the invasion of SKOV-3 cells.
Agarwal et al., 2010 (4)	MMP-1	MMP-1-PAR1 (protease-activated receptor-1) activation induces secretion of several angiogenic factors in ovarian carcinoma cells, leading to cell proliferation, tube formation and migration.

## Tissue inhibitors of matrix metalloproteinases (TIMPs)

**Figure 2.**
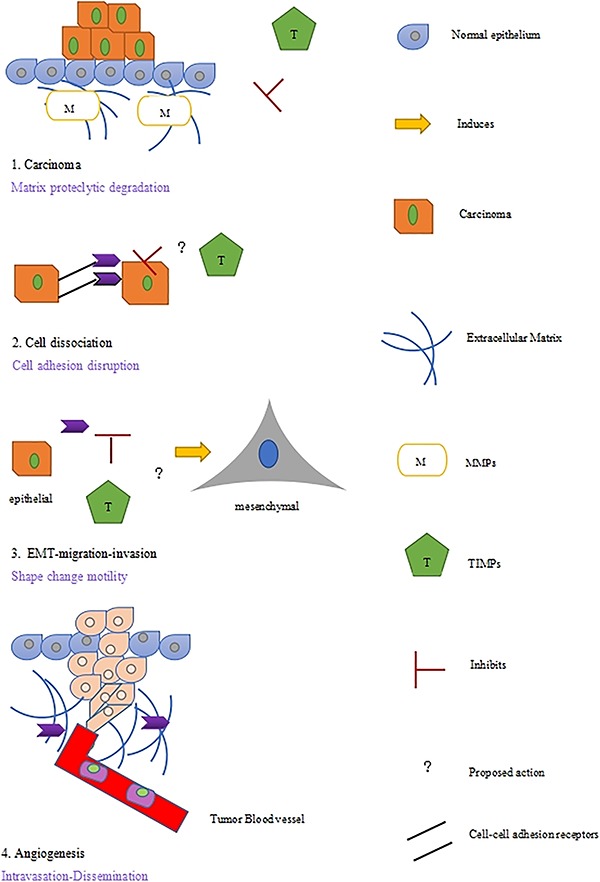
Matrix metalloproteinases (MMPs) and tissue inhibitors of matrix metalloproteinases (TIMPs) input in tumor cell adhesion during different levels of carcinoma progression. The proposed activities of TIMPs need further validation.

TIMPs play a central part in the regulation of MMPs activity (Figure 2). To date, four TIMPs have been found, namely TIMP-1, TIMP-2, TIMP-3 and TIMP-4. About 40% of the sequences of TIMPs molecules are homologous. TIMPs can inhibit the protease activity of MMPs by forming stable and irreversible non-covalent bonding with active MMPs in the ratio of 1:1. Some studies have shown that TIMPs play a critical role in the regulation of a wide range of cellular processes, including extracellular environment control, intercellular cell adhesion and signaling, such as growth factor signaling pathway, and ECM remodeling. TIMPs can interact with pro-MMPs to form stable complexes, and thus hinder pro-MMPs from further compounding with their substrates. Although TIMPs can inhibit all MMP molecules, some studies have suggested that they have specificity towards pro-MMPs: while pro-MMP-2 can interact with TIMP-2, TIMP-3 and TIMP-4, TIMP-2 has a higher specificity to MMP-2. MMP-9 can bind with TIMP-1 and TIMP-3, and TIMP1 can inhibit MMP-1, MMP-3 and MMP-9. Davidson et al. (40) found that an increased MMP-2 level and a decreased TIMP level in the exudation of the ovarian cancer cell might be used to mark the metastatic phenotypes. Hu et al. (41) observed that the expression of MMP-2, MMP-7, MMP-9, TIMP-2, and TIMP-3 in ovarian cancer is higher compared with normal ovarian tissues and benign ovarian tumors. They have also reported that overexpression of MMP-9 and the imbalance between MMP-9 and TIMP-1 play a significant role in the development of ovarian cancer. The increased serum level of TIMP-1 is also detected in ovarian cancer (42). Moreover, the increased expression of TIMPs suppresses the activity of MMPs and reserves the integrity of ECM (2). Gershtein et al. (43) found that serum levels of MMP-7, MMP-9 and TIMP-1 are positively correlated with the size of primary ovarian tumors. Imbalance between MMPs and TIMPs is closely related to the invasion and metastasis of ovarian cancer, despite that the underlying mechanisms need further investigation.

## Conclusion

Our research group has found that abnormal activation of hedgehog signaling pathway promotes the invasion and metastasis of ovarian cancer, which is mediated through the downstream target gene *MMP-7* (44). We suspect that the hedgehog signaling pathway regulates the MMP-7 expression on a transcriptional level by transcription factors GLI1/GLI2 binding to the promoter region of *MMP-7* although further investigation is needed.

In summary, MMPs are overexpressed in ovarian cancer, and significantly correlate with clinical stage, malignant degree, invasion and metastasis. Meanwhile, several studies have shown that TIMPs also play an important role in the invasion and metastasis of ovarian cancer. The imbalance between MMPs and TIMPs can facilitate the invasion and metastasis of ovarian cancer. MMPs and TIMPs may be used as sensitive biomarkers for the exploration of biological behaviors of ovarian cancer. Exploring the regulatory mechanism of MMPs and TIPMs would have remarkable clinical significance in alleviation of the occurrence, development, invasion and metastasis of ovarian cancer, which will benefit early diagnosis and treatment, improve the survival rate and reduce the mortality rate of the malignant disease.
